# Sofosbuvir add-on to ribavirin for chronic hepatitis E in a cirrhotic liver transplant recipient: a case report

**DOI:** 10.1186/s12876-019-0995-z

**Published:** 2019-05-24

**Authors:** Montserrat Fraga, Jérôme Gouttenoire, Roland Sahli, Haithem Chtioui, Cristina Marcu, Manuel Pascual, Darius Moradpour, Julien Vionnet

**Affiliations:** 1 Division of Gastroenterology and Hepatology, Centre Hospitalier Universitaire Vaudois, Rue du Bugnon 46, CH-1011 Lausanne, Switzerland; 2 Division of Gastroenterology and Hepatology, Institute of Microbiology, Lausanne, Switzerland; 3 Division of Clinical Pharmacology, Centre Hospitalier Universitaire Vaudois, University of Lausanne, Lausanne, Switzerland; 4 Transplantation Center, Centre Hospitalier Universitaire Vaudois, University of Lausanne, Lausanne, Switzerland; 5 Present address: Institute of Liver Studies, King’s College Hospital, London, UK

**Keywords:** Chronic hepatitis, Decompensated cirrhosis, Hepatitis E virus, Rabbit HEV, Solid organ transplantation

## Abstract

**Background:**

Chronic hepatitis E represents an emerging challenge in organ transplantation, as there are currently no established treatment options for patients who fail to clear hepatitis E virus (HEV) following reduction of immunosuppressive therapy and/or treatment with ribavirin. Sofosbuvir has shown antiviral activity against HEV in vitro but clinical utility in vivo is unknown.

**Case presentation:**

We describe a 57-year-old liver transplant recipient with decompensated graft cirrhosis due to chronic hepatitis E. Reduction of immunosuppressive treatment as well ribavirin alone for 4 months did not result in viral clearance. Add-on of sofosbuvir for 6 months was associated with HEV RNA becoming undetectable in plasma. However, sustained viral clearance could not be achieved.

**Conclusions:**

Sofosbuvir may have some antiviral activity against HEV when added to ribavirin. However, this did not suffice to yield sustained viral clearance. Our well-characterized observation emphasizes the need for new treatment options to cure chronic hepatitis E in the setting of organ transplantation.

## Background

Hepatitis E virus (HEV) is a leading cause of acute hepatitis and jaundice worldwide [1]. In low-income areas, HEV is transmitted via the fecal-oral route and associated with high morbidity and mortality, especially among pregnant women infected with HEV genotype 1. In middle- and high-income areas, HEV infection represents a zoonosis acquired primarily through the consumption of raw or undercooked pork or game meat. In this setting, HEV genotype 3 infection is, in most cases, responsible for self-limited disease. However, in the context of immunosuppression, chronic hepatitis can develop and evolve to cirrhosis in up to 10% of patients within a short period of 2 years.

Chronic HEV infection after transplantation is managed following a stepwise approach: First, immunosuppressive therapy is reduced if possible, resulting in viral clearance in about one third of cases. Second, ribavirin (RBV) monotherapy represents the current standard of care, resulting in viral clearance in 78% of patients treated for 12 weeks ([1] and references therein). Most patients eliminate HEV upon retreatment for a longer period. However, some patients do not achieve sustained viral clearance even after repeated and prolonged treatment courses or develop resistance to RBV. Novel antiviral strategies are needed for these patients.

Sofosbuvir (SOF), a highly potent hepatitis C virus (HCV) polymerase inhibitor, was shown to inhibit HEV genotype 3 replication in vitro, with an additive effect when combined with ribavirin [2]. Here, we report the outcome of SOF and RBV combination therapy in a liver transplant recipient with decompensated graft cirrhosis due to chronic hepatitis E and an insufficient response to RBV alone.

## Case presentation

A 57-year-old Caucasian male patient received a liver transplant in 1998 for alcoholic cirrhosis and hepatocellular carcinoma. In 2006, diffuse large B-cell lymphoma (post-transplant lymphoproliferative disease) was diagnosed and successfully treated with chemotherapy. The patient’s previous medical history also included psychiatric illness and post-traumatic epilepsy. His maintenance immunosuppressive treatment consisted of tacrolimus (trough levels 5–6 μg/l) and prednisone 5 mg qd.

Since 2014, routine control exams revealed slight intermittent transaminase elevation, attributed to suspected alcohol consumption. In August 2016, the patient presented with ascites and laboratory evidence of graft dysfunction (INR 1.3, albumin 34 g/l, total bilirubin 47 μmol/l, creatinine 99 μmol/l), without any signs of encephalopathy. Child-Pugh stage and MELD score were B9 and 14, respectively. Transaminases were moderately elevated (ALT 63 U/l, AST 110 U/l) and associated with some degree of cholestasis (alkaline phosphatase 240 U/l, γ-GT 502 U/l). Hepatitis B and hepatitis C as well as cytomegalovirus infections were ruled out by PCR. There was no significant increase in Epstein-Barr virus DNA which remained in the usual range for the patient (24,000 cp/ml).

Serology for both anti-HEV IgM and IgG was positive and so was PCR for HEV RNA in plasma (7.0 log_10_ IU/ml). Sequence analyses revealed infection with rabbit HEV (genotype 3ra) [3]. Positive HEV RNA could be found retrospectively in a stored serum sample from 2014, confirming the diagnosis of decompensated graft cirrhosis due to chronic hepatitis E.

**Fig. 1:**
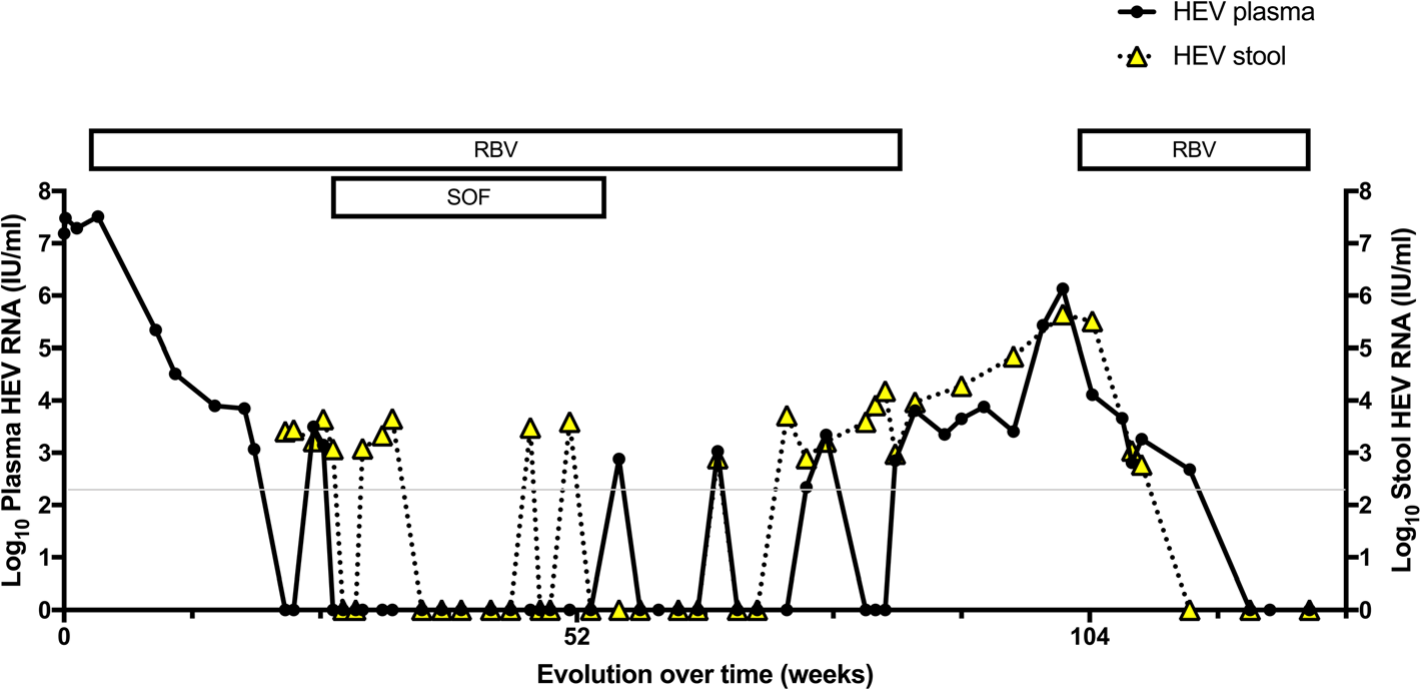
Evolution of hepatitis E virus (HEV) RNA in plasma (filled dots and lines) and stool (yellow triangles, dotted lines) over time. The thin grey line denotes the lower limit of detection of the PCR assay. RBV, ribavirin; SOF, sofosbuvir

Tacrolimus was reduced to yield trough levels around 2 μg/l, along with prednisone 5 mg qd. However, as HEV RNA did not decrease, RBV was introduced in September 2016, with trough levels between 1129 and 3700 ng/ml. Under this treatment, liver function tests normalized and there was a complete resolution of ascites. HEV RNA dropped but reached a plateau at 3 log_10_ IU/ml after 12–16 weeks of RBV therapy (Fig. 1). Thus, SOF 400 mg qd was added on a compassionate use basis from February to July 2017, i.e. for a total of 24 weeks.

Shortly after SOF introduction, HEV RNA became undetectable in plasma and remained so throughout the period of combination therapy (Fig. 1). Trough levels of the major SOF metabolite GS-331007 were in the expected concentration range for a patient with moderately impaired renal function (332–1966 ng/ml). HEV RNA in stool became negative 2 months after the introduction of SOF but a positive result was observed 2–3 months later, towards the end of combination therapy. In July 2017, SOF was stopped. Despite the maintenance of RBV, this resulted in the reappearance of HEV RNA in plasma and stool. After stop of RBV at the end of February 2018, HEV viremia remained relatively low for about 3 months (range, 3.7–4.8 log_10_ UI/ml) but increased again increased significantly to 6.1 log_10_ IU/ml in July 2018. Hence, RBV treatment was resumed in August 2018, with a slow decline in HEV RNA in plasma and stools, which both became undetectable at the end of November 2018, i.e. after more than 3 months (Fig. 1). The patient is now well and still under RBV treatment at the time of writing of this report in January 2019.

Sequencing of the polymerase region of open reading frame 1 in plasma samples obtained before (August 2016) and after RBV treatment (July 2018) revealed, as expected for rabbit HEV (genotype 3ra), a preexisting lysine in amino acid position 1634, which persisted throughout the observation period. Interestingly, among other amino acid changes observed, selection of an asparagine instead of a lysine was noted in position 1383 (K1382 N). Both the preexisting lysine in position 1634 and the selected asparagine in position 1383 had previously been identified in patients with RBV failure (reviewed in ref. [4])

To conclude, SOF appeared to exert some antiviral effect during combination therapy, resulting in negativation of HEV RNA in plasma. However, sustained viral clearance could not be achieved.

## Discussion and conclusion

There are currently no approved or recommended treatment options for solid organ transplant recipients with chronic hepatitis E who do not achieve sustained viral elimination after reduction of immunosuppressive treatment and prolonged or repeated courses of RBV [1]. Pegylated interferon-α was not considered to be an option in our patient. SOF exerts antiviral activity against HEV in vitro, which is, however, significantly lower than its activity against HCV [2]. Six cases involving the use of SOF in chronic hepatitis E have been reported so far, with mixed results: HEV clearance in two patients [5, 6], some antiviral effect but without sustained viral clearance in three others [7–9], and a lack of antiviral effect in one [10]. Of note, SOF was administered without RBV in the latter and the frequency of HEV RNA measurements was low.

In our well-characterized case report, including quantitative PCR for HEV RNA in plasma and stool, viral sequence analyses, as well as therapeutic drug monitoring for ribavirin and the major sofosbuvir metabolite GS-331007, SOF also appeared to have some antiviral effect against HEV when added to RBV. However, this was not sufficient to yield sustained viral clearance, as presaged by the reappearance of HEV RNA in stool towards the end of antiviral combination therapy [1]. Clearly, more studies are required to draw firm conclusions on any clinically useful activity of SOF against HEV in vivo.

In conclusion, this clinical case emphasizes the difficulty of treating patients chronically infected with HEV in the setting of an insufficient response to RBV. The burden of chronic hepatitis E is probably still underestimated and clinicians will likely face growing numbers of such difficult-to-treat cases. Given the rapid progression to cirrhosis, new treatment options for chronic hepatitis E should be prospectively studied to improve its management.

## Abbreviations

HCV: Hepatitis C virus
HEV: Hepatitis E virus
RBV: Ribavirin
SOF: Sofosbuvir
